# Electroconvulsive therapy knowledge and attitudes among patients and caregivers in South China: A preliminary study

**DOI:** 10.3389/fpsyt.2023.1145301

**Published:** 2023-03-13

**Authors:** Can-Jin Deng, Sha Nie, Jian-Xin Mai, Xiong Huang, Xing-Bing Huang, Wei Zheng

**Affiliations:** The Affiliated Brain Hospital of Guangzhou Medical University, Guangzhou, China

**Keywords:** electroconvulsive therapy, attitudes, knowledge, caregivers, major psychiatric disorders

## Abstract

**Background:**

Electroconvulsive therapy (ECT) is a safe and effective therapy for individuals suffering from major psychiatric disorders, but attitudes towards ECT among patients and caregivers have not been well studied. This study was conducted to elucidate patient and caregiver knowledge and attitudes concerning ECT in South China.

**Methods:**

The sample comprised 92 patients diagnosed with major psychiatric disorders and their caregivers (*n* = 92). Participants completed questionnaire measures of knowledge and attitudes related to ECT.

**Results:**

Information before ECT was inadequately provided to both caregivers and patients (55.4% versus 37.0%, *p* < 0.05). Caregivers reported receiving more adequate information about the therapeutic effects (50.0% versus 44.6%), side effects (67.4% versus 41.3%), and risks (55.4% versus 20.7%) of ECT when compared to patients (all *p* < 0.05). However, less than half of patients and caregivers believed that ECT was effective (43.5% versus 46.7%, *p* > 0.05), while more than half of them believed that ECT was beneficial (53.3% versus 71.7%, *p* < 0.05), and approximately half of them believed that ECT was safe (50.0% versus 51.1%, *p* > 0.05). A total of 32.6% of patients and 55.4% of caregivers (*p* < 0.05) reported that ECT was used only for critically ill patients. A total of 62.0% of patients experienced side effects, with memory impairment being the most commonly reported.

**Conclusion:**

Clinicians should develop a systematic health education program before ECT treatment and ensure that patients and caregivers have an accurate understanding of ECT, particularly the treatment process, its therapeutic effects and potential side effects prior to administering this treatment.

## 1. Introduction

Electroconvulsive therapy (ECT), as a non-invasive brain stimulation technique (1), is effective in treating severe psychiatric disorders such as bipolar disorder (BD), schizophrenia, and major depressive disorder (MDD) in clinical practice (2–5). Since its introduction as a non-invasive brain stimulation technique in 1938, ECT has been widely used in many countries, including the United States (5, 6) and China (7, 8). Apart from ECT, other non-invasive brain stimulation techniques, such as repetitive transcranial magnetic stimulation (rTMS), transcranial direct current stimulation (tDCS), and transcranial alternating current stimulation (tACS) have been widely used in treating major mental disorders (6). ECT has been shown to be more effective than rTMS, tACS, or tDCS for treatment-resistant depression (TRD) (5, 9).

In general, ECT is a highly effective treatment for severe psychiatric disorders, and the gold-standard therapy for patients suffering from TRD (10). While ECT is a therapeutically effective alternative in older patients and adolescents with major psychiatric disorders (11, 12), some studies have discouraged the use of ECT because of its potential adverse effects, such as short-term confusion and memory impairment (13). For example, a meta-analysis of randomized controlled studies (RCTs) found that ECT combined with antipsychotics led to significantly greater transient memory impairment in patients with schizophrenia (13).

The number of patients who have received a course of ECT is vast in China. For example, a cross-sectional survey (*n* = 1,364) focusing on adult patients (≥18 years old) found that 52.1% received ECT, with 53.4% receiving ECT for MDD, 57.8% for BD, 57.0% for schizophrenia, and 32.4% for other diagnoses (14). In a retrospective study, 28.1% of 2,339 hospitalized older Chinese psychiatric patients (aged 60 years and older) received ECT, including 21.2% for schizophrenia, 43.6% for MDD, 37.9% for BD, and 10.7% for other diagnoses (15). Another retrospective study focusing on Chinese adolescent psychiatric patients aged between 13 and 17 years found that 42.6% of the total sample received ECT, including 57.8% for BD, 41.8% for MDD, 46.5% for schizophrenia, and 23.9% for other diagnoses (16). However, there is an ongoing debate about the use of ECT for major psychiatric disorders in China. Some advocate its use because of rapid improvements in psychiatric symptoms (17), while others argue that the utility of ECT is limited given its adverse effects (18).

To understand the acceptance of ECT as a treatment among patients with psychiatric disorders who had ECT, it is important to investigate patient and caregiver attitudes and knowledge regarding the intervention (19). Relatively few studies have examined this issue (19), and related findings have been inconsistent (7, 19–21). For example, a previous study found that caregivers (75.0%) reported receiving significantly more adequate information about the therapeutic effects of ECT than did older psychiatric patients (56.1%) (19). However, another study found that more adolescents with psychiatric disorders (55.7%) reported receiving adequate information about the therapeutic effects of ECT than their caregivers (50.6%) (20). Given that ECT practice is influenced by cultural, social, and legal factors, findings from previous studies on ECT attitudes and knowledge from other countries or regions may not be applicable to samples in different regions of China (22–24).

Thus, the purpose of this study was to investigate the knowledge and attitudes towards ECT among patients and caregivers as well as patients’ subjective experiences regarding ECT in South China.

## 2. Methods

### 2.1. Study design and participants

This cross-sectional survey was conducted from 5 February 2021 to 31 May 2022 at the Affiliated Brain Hospital of Guangzhou Medical University, a setting that has 1800 psychiatric beds and performs approximately 1,500 ECT sessions monthly. The study protocol was approved by the Research Ethics Panel of the Affiliated Brain Hospital of Guangzhou Medical University (No. 2021001). Both patients receiving a course of ECT sessions and caregivers who provided informed consent in writing were invited to join the study.

The following were inclusion criteria for patients in this study: (1) understanding the questionnaire content focusing on knowledge and attitudes towards ECT; (2) a diagnosis of schizophrenia, BD, or MDD; (3) aged 18 years or older; (4) treated with a course of ECT in the past 6 months; and (5) completion of the last ECT session more than 1 month before this questionnaire. Similarly, caregivers had to fulfill the following inclusion criteria: (1) understanding of the questionnaire content; (2) aged 18 years or older; and (3) a friend or family member (e.g., spouse, sister, and brother) who has provided primary personal care and handled medical-related issues for the patient during the past 6 months.

### 2.2. ECT parameters

ECT is available only as an inpatient procedure at the Affiliated Brain Hospital of Guangzhou Medical University. ECT (Spectrum 5000Q ECT machine, MECTA Inc., Lake Oswego, OR, USA) generally consists of 6–12 sessions per course, performed 2–3 times per week from 8:00 a.m. to noon on weekdays. During each ECT session, the patient’s blood oxygen, heart rate, and blood pressure are monitored with a cardiac monitor.

### 2.3. Measures

Face-to-face interviews were performed by one interviewer for all assessments. According to the methodology of previous studies (7, 19, 21), patients completed a three-part self-report questionnaire that included subjective experience, knowledge, and attitudes towards ECT. Another knowledge and attitudes questionnaire about ECT was given to caregivers. Each question on the questionnaire offered three options: “I do not know,”"disagree/no,” and “agree/yes.”

### 2.4. Statistical analysis

All statistical analyses in the present study were performed using SPSS version 24.0 (SPSS Inc., Chicago, IL, USA). For descriptive analyses, continuous and categorical variables were expressed as the means ± standard deviations (SD) and percentages (%) with regard to the demographic and clinical data of patients. Chi-square tests were utilized to determine the differences in ECT knowledge and attitudes between patients with major psychiatric disorders and their caregivers. *p* values less than 0.05 (two-tailed) were deemed statistically significant.

## 3. Results

### 3.1. Basic demographic and clinical data

Of the 96 patients and 96 caregivers invited to participate in the study, 92 (95.8%) patients with major psychiatric disorders who received ECT and 92 (95.8%) caregivers fulfilled the inclusion criteria and completed the assessment. Demographic and clinical data of subjects undergoing ECT are summarized in Table 1. Among the patients, 16.3%, 52.2%, and 31.5% were diagnosed with schizophrenia, BD, and MDD, respectively; 57.6% of patients were female, and 32.6% were married. The mean age, age of onset, and body mass index (kg/m^2^) of patients were 27.9 ± 10.7 years, 20.9 ± 7.5 years, and 22.3 ± 2.6 kg/m^2^, respectively.

**Table 1:** Basic demographic and clinical characteristics of patients receiving electroconvulsive therapy.

Variables	Patients (*n* = 92)	
	*n*	%
Female	53	57.6
Married	30	32.6
Employed Full-time	28	30.4
Principal diagnosis		
Schizophrenia	15	16.3
Bipolar disorder	48	52.2
Major depressive disorder	29	31.5
Past ECT treatment	26	28.3
	Mean	SD
Age (years)	27.9	10.7
Age of onset(years)	20.9	7.5
Number of hospitalizations	3.4	3.2
Number of ECTsessions during the recent hospitalization	7.1	2.1
BMI (kg/m2)	22.3	2.6
Education(years)	12.0	3.6

ECT: electroconvulsive therapy; BMI: body mass index.

### 3.2. ECT knowledge of patients and caregivers

As shown in Table 2, 55.4% of caregivers and 37.0% of patients reported that they received any information before ECT (*p* < 0.05). Caregivers (versus patients) reported receiving relatively more adequate information regarding therapeutic effects (50.0% versus 44.6%), side effects (67.4% versus 41.3%), and risks (55.4% versus 20.7%) of ECT (all *p* < 0.05). However, patients (versus caregivers) reported receiving more adequate information on the process of ECT (45.7% versus 23.9%) (*p* < 0.05). A significantly larger percentage of caregivers (71.7%) versus patients (53.3%) believed that ECT was beneficial (p < 0.05). Approximately half of patients (versus caregivers) thought that ECT was more effective (43.5% versus 46.7%) and more rapid than drugs (51.1% versus 45.7%) (all *p* > 0.05).

**Table 2:** Electroconvulsive therapy knowledge and perceptions of patients and their caregivers.

Questionnaire items	Patients (*n* = 92)						Caregivers (*n* = 92)						Statistics	
	Disagree		Agree		Do not know		Disagree		Agree		Do not know			
	*n*	%	*n*	%	*n*	%	*n*	%	*n*	%	*n*	%	*X* ^2^	*p*
Were you given any information before ECT?	38	41.3	34	37.0	20	21.7	30	32.6	51	55.4	11	12.0	7.0	**0.03**
Were you given adequate information concerning the therapeutic effects of ECT?	26	28.3	41	44.6	25	27.2	35	38.0	46	50.0	11	12.0	7.1	**0.03**
Were you given adequate information concerning the process of ECT?	23	25.0	42	45.7	27	29.3	58	63.0	22	23.9	12	13.0	27.1	**<0.001**
Were you given adequate information concerning the sideeffects of ECT?	26	28.3	38	41.3	28	30.4	20	21.7	62	67.4	10	10.9	15.1	**0.001**
Were you given adequate information concerning the risks of ECT?	36	39.1	19	20.7	37	40.2	25	27.2	51	55.4	16	17.4	24.9	**<0.001**
Do you think health professionals provided adequate information about ECT?	45	48.9	21	22.8	26	28.3	16	17.4	68	73.9	8	8.7	48.1	**<0.001**
Do you feel ECT has been beneficial?	20	21.7	49	53.3	23	25.0	16	17.4	66	71.7	10	10.9	8.1	**0.02**
Do you feel ECT has made you/your relative worse?	36	39.1	24	26.1	32	34.8	43	46.7	25	27.2	24	26.1	1.8	0.41
Do you feel ECT has been more effective than drugs?	18	19.6	40	43.5	34	37.0	17	18.5	43	46.7	32	34.8	0.2	0.91
Do you think the effect of ECT is more rapid than drugs?	12	13.0	47	51.1	33	35.9	20	21.7	42	45.7	30	32.6	2.4	0.30

ECT: electroconvulsive therapy; *p* < 0.05 is in bold.

### 3.3. ECT attitudes of patients and caregivers

As depicted in Table 3, 50.0% of patients and 51.1% of caregivers reported positive attitudes towards ECT (*p* > 0.05). More patients than caregivers disagreed with the statement, “Do you think ECT is dangerous and should not be used?” (66.3% versus 47.8%), and a significantly higher proportion of caregivers than patients endorsed the following queries: “Should ECT be used only for critically ill patients?” (55.4% versus 32.6%), “Should ECT be the last resort?” (51.1% versus 23.9%), and “Would you/your relative like to receive ECT again?” (83.7% versus 55.4%) (all *p* < 0.05). Alternatively, a significantly higher proportion of patients (31.5%)versus caregivers (12.0%) responded affirmatively to the question, “Is ECT used for people who do not need it?” (p < 0.05).

**Table 3:** Attitudes toward electroconvulsive therapy among patients and their caregivers.

Questionnaire items	Patients (*n* = 92)						Caregivers (*n* = 92)						Statistics	
	Disagree		Agree		Do not know		Disagree		Agree		Do not know			
	*n*	%	*n*	%	*n*	%	*n*	%	*n*	%	*n*	%	*X* ^2^	*p*
Is ECT safe?	15	16.3	46	50.0	31	33.7	23	25.0	47	51.1	22	23.9	3.2	0.20
Do you fear ECT?	46	50.0	24	26.1	22	23.9	51	55.4	29	31.5	12	13.0	3.7	0.16
Do you think ECT is more dangerous than drugs?	47	51.1	29	31.5	16	17.4	36	39.1	34	37.0	22	23.9	2.8	0.25
Do you think ECT is dangerous and should not be used?	61	66.3	12	13.0	19	20.7	44	47.8	25	27.2	23	25.0	7.7	**0.02**
Is ECT used for people who do not need it?	28	30.4	29	31.5	35	38.0	44	47.8	11	12.0	37	40.2	11.7	**0.003**
Should ECT be used only for critically ill patients?	35	38.0	30	32.6	27	29.3	16	17.4	51	55.4	25	27.2	12.6	**0.002**
Should ECT be the last resort?	45	48.9	22	23.9	25	27.2	22	23.9	47	51.1	23	25.0	17.0	**<0.001**
Is ECT used to punish patients?	66	71.7	9	9.8	17	18.5	74	80.4	8	8.7	10	10.9	2.3	0.31
Would you/your relative like to receive ECT again?	29	31.5	51	55.4	12	13.0	7	7.6	77	83.7	8	8.7	19.5	**<0.001**

ECT: electroconvulsive therapy; *p* < 0.05 is in bold.

### 3.4. Patient experiences of ECT and associated side effects

Patient experiences of having ECT are summarized in Table 4. During ECT, up to 76.1% of patients felt worried, particularly when receiving an intravenous injection (46.7%) or when thinking about receiving a treatment that used electricity (28.3%). As shown in Table 5, 62.0% of patients reported side effects caused by ECT; within this group, memory impairment (72.8%) was the most common side effect, followed by headache (46.7%) and short-term confusion (45.7%).

**Table 4:** Patient worries about receiving electroconvulsive therapy.

Areas of concern	Patient Responses (*n* = 92)			
	Yes		No	
	*n*	%	*n*	%
Did you feel worried?	70	76.1	22	23.9
Specific concerns				
Waiting for the treatment	23	25.0	69	75.0
Wearing an oxygen mask	16	17.4	76	82.6
Wearing a headband	8	8.7	84	91.3
Receiving an intravenous injection	43	46.7	49	53.3
Being unconscious	25	27.2	67	72.8
Waking up after the treatment	15	16.3	77	83.7
Thinking about receiving a treatment using electricity	26	28.3	66	71.7

**Table 5:** Patient experiences of electroconvulsive therapy adverseeffects.

Questionnaire items	Patients (*n* = 92)					
	Disagree		Agree		Do not know	
	*n*	%	*n*	%	*n*	%
Have you had any adverse effects?	17	18.5	57	62.0	18	19.6
Did you have a headache?	42	45.7	43	46.7	7	7.6
Did you have muscle pain?	60	65.2	27	29.3	5	5.4
Did you have a poor appetite or nausea?	68	73.9	14	15.2	10	10.9
Did you have short-term confusion?	35	38.0	42	45.7	15	16.3
Did you have memory impairment?	17	18.5	67	72.8	8	8.7

## 4. Discussion

As reported by Tang et al. (8), China has the largest patient population receiving ECT in the world. The main findings of this study were: (1) patients and caregivers were not adequately provided with comprehensive information about ECT yet generally had positive attitudes towards ECT; (2) patients reported receiving an intravenous injection as their most pressing worry during ECT; and (3) patients who had side effects endorsed memory impairment as the most common side effect of ECT.

In this study, only 37.0% of patients and 55.4% of caregivers reported receiving any information about ECT before ECT was conducted. Similar findings from samples in Hong Kong, China (19.8% in patients and 58.6% in caregivers) (21), Beijing, China (42.4% in patients and 59.5% in caregivers) (7), and Turkey (32.9% in patients and 51.4% in caregivers) (25) have been reported previously. Furthermore, substantial percentages of patients and caregivers reported not being informed about the process of ECT, therapeutic effects, and risks of ECT, consistent with low rates reported by previous studies (7, 19). Patients have generally positive attitudes toward rTMS (6), while no studies that examine the knowledge and attitudes of other non-invasive brain stimulation techniques including tACS, tDCS or magnetic seizure therapy (MST) among patients and caregivers have been published. To improve the knowledge of patients and caregivers toward ECT or other non-invasive brain stimulation techniques, systematic health education methods, such as video, brochures and video-assisted educational programs, need to be developed (26–28), and follow-ups are needed to ensure that patients and caregivers have an accurate understanding of the information they have received. Patients reported lower ECT knowledge levels than their caregivers (7, 19, 21), which may be partly attributed to illness factors, such as ECT-induced memory impairment and inadequate or lack of insight about their condition (7, 29, 30). However, the cognition functions and insight recovery of patients were unclear because they were not assessed in this study.

In the past, ECT was often misrepresented in the mass media as a punitive measure for aggressive behaviour; such portrayals contributed to negative public attitudes towards ECT (31, 32). Encouragingly, our study showed that approximately half of patients and their caregivers had positive attitudes towards ECT, viewing it as a beneficial and more effective and rapid treatment than drugs. Furthermore, approximately half of patients and caregivers believed that ECT is safe (50.0% versus 51.1%) and does not induce fear (50.0% versus 55.4%). Approximately three-quarters of patients (71.7%) and caregivers (80.4%) believed that ECT is not a punishment. Up to 83.7% of caregivers and 55.4% of patients might have received ECT again if necessary. Taken together, these findings in the current study do not support the negative attitudes regarding ECT in China (8, 21). Unlike in many Western countries, ECT is widely accepted by clinicians and patients in China, and it has been frequently prescribed in clinical practice (33). For example, a recent study found that both older Chinese patients with severe psychiatric disorders and their caregivers mostly reported positive attitudes towards ECT (19). The possible reasons for the above inconsistent findings were mainly attributed to the fact that the use of ECT is associated with cultural, social, and legal factors (22–24).

A majority of patients (76.1%) reported feeling worried during ECT, which is similar to findings of previous studies (7, 19). However, the incidence of worry has varied widely across studies, ranging from 14% to 75% (34, 35). The most commonly reported worry in our study was receiving intravenous injections, in contrast to other studies that have reported memory impairment or brain damage as the most pressing worries (36). Higher levels of ECT knowledge are related to lower levels of fear and more positive attitudes towards ECT (37). Consequently, psychiatric clinicians should educate patients and caregivers about ECT, including its nature and side effects. As part of the process, patient understanding of information should also be assessed.

Approximately two-thirds of the patients experienced side effects of ECT, including memory impairment (72.8%), short-term confusion (45.7%), and headache (46.7%). Memory impairment was the most commonly reported side effect, while associated rates have varied in the literature from 29% to 79% (38–40). More severe side effects may be associated with higher doses of ECT, existing brain disease, and older age (16, 41). A recent systematic review found that nonconvulsive electrotherapy (NET) appeared to be a safe and effective treatment option for patients diagnosed with depression without serious cognitive impairments (30). Furthermore, a recent meta-analysis found that MST showed shorter recovery time and reorientation time when compared to ECT (42). However, no head-to-head studies have compared the effectiveness and cognitive effects of NET and MST in severe psychiatric disorders, especially MDD.

The main strength of this study is the first to assess ECT knowledge and attitudes among patients who had undergone the intervention and their caregivers in South China. However, this study has several limitations that should be acknowledged. First, there is no standardized preexisting, interviewer-rated tool to assess ECT knowledge and attitudes. Second, this study with a single-center design was conducted in a psychiatric hospital in southern China. Therefore, the findings of this study cannot be generalized to other countries. Third, the sample size of this study was relatively small, limiting our capacity to detect the differences in attitudes or experiences of ECT among patients with different diagnoses. Fourth, key factors influencing participants’ attitudes towards ECT, such as disease severity and the nature/use of psychiatric medications, were not evaluated. Finally, this cross-sectional study has not been registered.

In conclusion, clinicians should develop a systematic health education program before ECT treatment and ensure that patients and their caregivers have an accurate understanding of ECT, particularly the treatment process, its therapeutic effects and potential side effects prior to administering this treatment.

## Data availability statement

The raw data supporting the conclusions of this article will be made available by the authors, without undue reservation.

## Ethics statement

The studies involving human participants were reviewed and approved by the Research Ethics Panel of the Affiliated Brain Hospital of Guangzhou Medical University. The patients/participants provided their written informed consent to participate in this study.

## Author contributions

WZ was responsible for study design, process supervision, data interpretation, and critical revision of the article. SN and C-JD were responsible for organizing and analyzing the data, data interpretation, and drafting the manuscript. J-XM, XH, and X-BH were responsible for implementing the study, data collection, and data interpretation. All authors contributed to the article and approved the submitted version.

## Funding

This study was funded by Guangzhou Liwan District Science and Technology Plan Project (202201012), Guangzhou Science and Technology Project of Traditional Chinese Medicine and integrated traditional Chinese and Western medicine (20211A011045), China International Medical Exchange Foundation (Z-2018-35-2002), Guangzhou Clinical Characteristic Technology Project (2019TS67), and Science and Technology Program Project of Guangzhou (202102020658). The funders had no role in study design, data collection, and analysis, decision to publish, or preparation of the manuscript.

## Conflict of interest

The authors declare that the research was conducted in the absence of any commercial or financial relationships that could be construed as a potential conflict of interest.

## Publisher’s note

All claims expressed in this article are solely those of the authors and do not necessarily represent those of their affiliated organizations, or those of the publisher, the editors and the reviewers. Any product that may be evaluated in this article, or claim that may be made by its manufacturer, is not guaranteed or endorsed by the publisher.
